# Radiation safety in catheter ablation: clinical value of real-time operator dosimetry

**DOI:** 10.1007/s00380-025-02567-x

**Published:** 2025-06-23

**Authors:** Machiko Miyoshi, Kanae Hasegawa, Rikiya Maruyama, Toshiki Tateishi, Ryohei Nomura, Toshihiko Tsuji, Moe Mukai, Tetsuya Tsujikawa, Hiroyasu Uzui, Hiroshi Tada

**Affiliations:** 1 Department of Cardiovascular Medicine, Faculty of Medical Sciences, University of Fukui, 23-3, Matsuokashimoaizuki, Eiheiji-cho, Yoshida-gun, Fukui, 910-1193 Japan; 2 Department of Radiology, Faculty of Medical Sciences, University of Fukui, Fukui, Japan

**Keywords:** Catheter ablation, Dosimeter, Radiation exposure, Radiation dose, Real-time radiation monitoring

## Abstract

Advances in interventional cardiology have increased procedural complexity, raising concerns about radiation exposure—especially for women operators of reproductive age, who are subject to stricter dose limits. Standard dosimeters provide only delayed cumulative data, limiting timely risk assessment. Real-time personal dosimetry offers immediate feedback, but its clinical utility during catheter ablation (CA) remains underexplored. We retrospectively analyzed 82 CA procedures performed between January and May 2024. First operators wore real-time dosimeters positioned at the waist under lead aprons. Radiation exposure and procedural characteristics were recorded and analyzed. The median operator radiation dose per procedure was 2 [1, 3] µSv, while the median patient dose was 0.163 [0.082, 0.324] Gy. A procedure-related cardiac tamponade requiring pericardiocentesis resulted in the highest operator dose (50 µSv). Higher patient BMI (≥ 25 kg/m^2^) and longer fluoroscopy time were independently associated with increased operator exposure (OR: 1.238, P = 0.008; OR: 1.056, P = 0.022), though no linear correlation was observed between BMI and operator dose (r = 0.029, P = 0.797). Radiation exposure to operators during CA is generally low but may increase significantly in the event of complications or with higher-risk patient characteristics. Real-time dosimetry provides valuable immediate feedback and may be especially important for radiation-sensitive operators, supporting safer practice in the evolving field of interventional electrophysiology.

## Introduction

Advances in technology have significantly impacted interventional cardiology, leading to both increased procedural complexity and efforts to minimize radiation exposure. While some studies suggest that radiation doses in interventional cardiology are rising due to prolonged procedures and increased use of fluoroscopy [1], other reports indicate that advances in radiation protection and imaging techniques have contributed to dose reduction [2]. Therefore, a balanced perspective is required when assessing radiation exposure trends in catheterization laboratories.

The number of interventional cardiology procedures, particularly catheter ablation (CA), has been increasing rapidly [3]. Radiation exposure remains a critical concern, especially for women cardiologists, given their unique occupational risks and potential reproductive concerns [4–6]. Despite the requirement for all catheterization laboratory personnel to wear dosimeters to monitor radiation exposure [7], standard dosimeters only provide cumulative exposure data retrospectively, often with a delay of several months. This lack of real-time monitoring may hinder immediate awareness and timely implementation of radiation reduction strategies.

Real-time personal dosimetry has emerged as a potential solution to address this issue, offering instant feedback and enabling operators to adjust their techniques to minimize radiation exposure. However, comprehensive reports on personal real-time dosimetry during CA are still lacking. We aimed to assess whether real-time dosimetry can offer meaningful and timely feedback to support radiation safety for operators, especially women of reproductive age, participating in interventional procedures.

Therefore, this study aimed to evaluate the factors contributing to the first operator's radiation exposure during CA using real-time personal dosimetry, with a focus on patient and procedural characteristics that may influence the radiation dose.

## Methods

This retrospective study included consecutive 82 CAs performed between January and May 2024 at the University of Fukui. All first operators wore personal dosimeters (Mydose Mini PDM-117, ALOKA, Hitachi, Japan) [8–10] which could measure radiation exposure in 1 μSv increments, at the waist under a lead apron to measure the radiation exposure dose per procedure. All operators were instructed to use ceiling-mounted upper body shield whenever possible. A 3D mapping system was used in all ablation procedures, except for typical common atrial flutter cases. Intracardiac echocardiography (ICE) was used in all atrial fibrillation (AF) ablation procedures and in cases of ventricular arrhythmias originating from the left ventricle. In addition, biplane fluoroscopy was used for all procedures. The frame rates for fluoroscopy and cine-fluoroscopy were set at 3.5 frames per second (fps) and 7.5 fps, respectively. Patient radiation dose was measured using the dose area product (DAP), which was automatically recorded by the fluoroscopy system (Artis zee or Artis Q, Siemens Healthcare, Germany) for each procedure. The DAP value (in Gy·cm^2^) reflects the total radiation energy delivered to the patient, considering both the dose and the area exposed.

Five first operators performed CA; 60% of them were women in their reproductive years. Procedural data, including the types of arrhythmias targeted for CA and radiation exposure for operators and patients, were analyzed and compared.

All patients provided written informed consent for the procedure, and the study protocol was approved by the Research Ethics Committee of the University of Fukui (20160040). Data supporting the findings of this study are available from the corresponding author upon reasonable request. The study complied with the Declaration of Helsinki.

### Statistical analysis

Continuous variables are presented as medians and interquartile ranges (IQR) and categorical data as numbers and percentages. Univariate analyses of categorical variables were performed using Fisher’s exact test. To avoid the assumption of normality, univariate analyses of continuous variables were performed using the Wilcoxon rank-sum test. To determine factors associated with radiation exposure to the operator ≥ 2 µSv, multivariable logistic regression was performed after adjustment for age, sex, body mass index (BMI), operative time, fluoroscopy time, right jugular vein puncture, and atrial fibrillation (AF). All statistical analyses were performed using SPSS software, version 26 (IBM Corp., Armonk, NY, USA).

## Results

### Baseline patient and procedural characteristics

A total of 82 patients who underwent CAs were included in this study (Table 1). Most arrhythmias indicated for CA were AF (72.8%) (Table 2). The radiation exposure to the patient was 0.163 [0.082, 0.324] Gy, and to the operator during CA was 2 [1, 3] µSv (Table 1). The radiation dose from left anterior oblique view and right anterior oblique or anteroposterior view was 0.087 [0.035, 0.188] and 0.079 [0.038, 0.135] Gy, respectively (P = 0.399), indicating no significant difference between the two groups (Table 1).

**Table 1:** Characteristics and procedure data in this cohort

N	82
Age, years	69 [60, 76]
Male, n (%)	50 (61.0)
Body mass index, kg/m^2^	23.2 [20.2, 26.3]
Operative time, min	178 [134, 220]
Fluoroscopy time, min	49 [28, 56]
Radiation dose for the patient, Gy	0.163 [0.082, 0.324]
From left anterior oblique view, Gy	0.087 [0.035, 0.188]
From right anterior oblique or anteroposterior view, Gy	0.079 [0.038, 0.135]
Radiation dose for the first operator, μSv	2 [1, 3]

Values are expressed as n (%) or medians [first and third quartiles]

**Table 2:** Targeting arrhythmia for catheter ablation

	N (%)
Atrial arrhythmias	64 (79.0)
Atrial fibrillation	59 (72.8)
Atrial flutter	3 (3.7)
Atrial tachycardia	2 (2.4)
Ventricular arrhythmias	12 (14.8)
Premature ventricular contraction	9 (11.1)
Ventricular tachycardia	3 (3.7)
Paroxysmal supraventricular tachycardia	6 (7.3)
AV nodal reentrant tachycardia	4 (4.9)
AV reciprocating tachycardia	2 (2.4)

Values are expressed as n (%)

*AV* atrio-ventricular

**Fig. 1:**
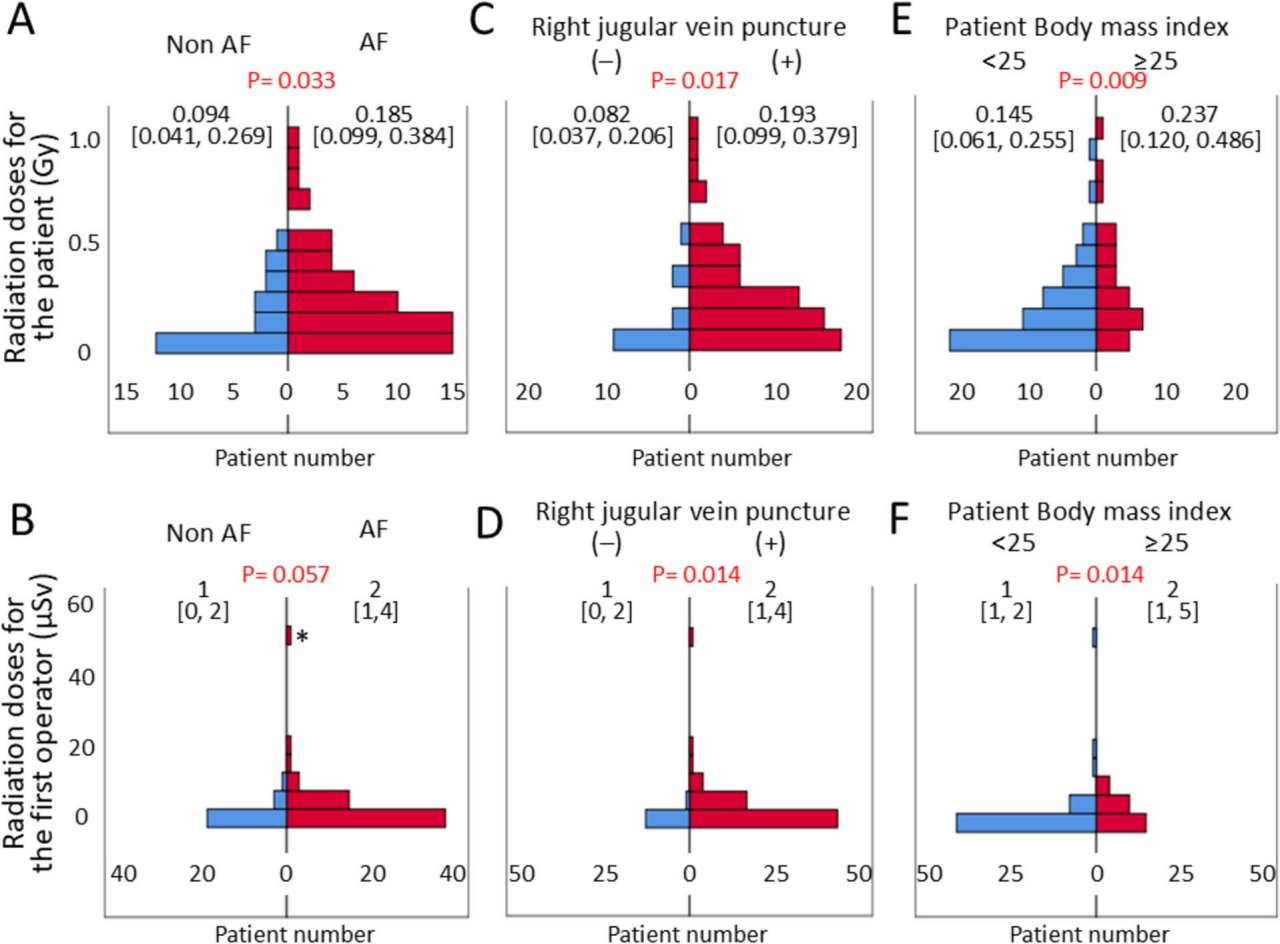
Comparison of radiation doses to the patient and the first operator during catheter ablation. **A**, **B** Targeted arrhythmias for atrial fibrillation (AF) and non AF. **C**, **D** In patients with and without additional right jugular vein puncture. **E**, **F** According to patients’ body mass index

The radiation exposure to patients was higher during CA for AF than during CA for non AF (Fig. 1A). The radiation exposure to the operator during CA was not significantly different between for AF and non-AF (Fig. 1B). Regarding CA for AF, the radiation exposure to the patient and operator was not significantly different between procedures using cryoballoon catheter and only radiofrequency catheter (0.202 [0.097, 0.392] vs 0.145 [0.108, 0.217] Gy, P = 0.405, and 2 [1, 3] vs 2 [1, 5] μSv, P = 0.940, respectively). The highest radiation exposure to the operator (indicated by an asterisk in Fig. 1B) was 50 μSv during CA for AF in a 61 year-old woman. In this patient, after pulmonary vein isolation using a cryoballoon, mitral isthmus-dependent atrial tachycardia and common atrial flutter were induced, and mitral and cavotricuspid isthmus block lines were created using a radiofrequency catheter. Immediately after confirmation of both block lines, the patient developed cardiac tamponade and required pericardiocentesis using fluoroscopy-guided technique in posterior-anterior and lateral fluoroscopies. No intraoperative complications were observed in any other patient.

Sixty-eight patients (82.9%) underwent right jugular vein puncture in addition to femoral puncture. Among them, 52 underwent CA for AF. Right jugular vein puncture increased the radiation exposure to the patient and operator (Fig. 1C, D).

### Effect of patient BMI on radiation exposure during CA

**Fig. 2:**
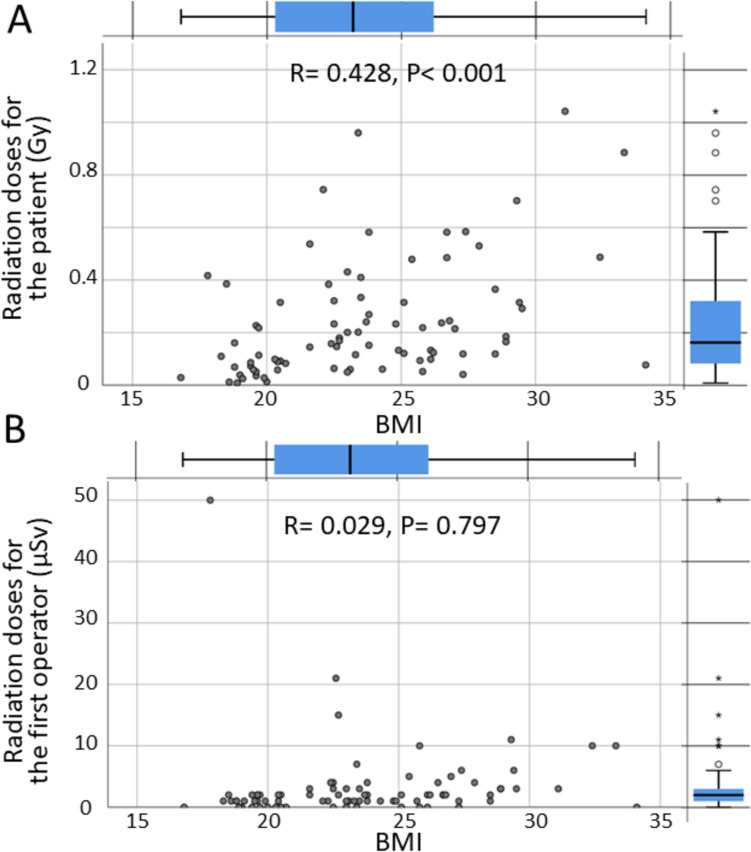
Relationship between exposure dose and body mass index during catheter ablation. **A** Exposure dose of the patient. **B** Exposure dose of the first operator

The radiation exposure to the patient and operator was higher when treating patients with BMI ≥ 25 kg/m^2^ than when treating patients with BMI < 25 kg/m^2^ (0.237 [0.020, 0.486] vs. 0.145 [0.061, 0.255] Gy, P = 0.009, and 2 [1, 5] vs. 1 [1, 2] μSv, P = 0.014, respectively) (Fig. 1E, F). Though BMI was correlated with the radiation exposure to the patients (r = 0.428, P < 0.001), no linear correlation was observed between BMI and radiation exposure to the operator (r = 0.029, P = 0.797) (Fig. 2A, B).

### Predictors for radiation exposure to the operator during CA

To evaluate the risk factors for higher exposure to the operator, a median radiation exposure of 2 μSv was set as the cutoff value. In the multivariable model, the predictors for radiation doses to the operator ≥ 2 µSv were higher patient BMI (OR, 1.238; P = 0.008), and longer fluoroscopy time (OR, 1.056; P = 0.022) (Table 3).

**Table 3:** Multivariable model for risk for radiation doses ≥ 2 µSv for the first operator

	Odds ratio	95% CI		P value
Age	0.997	0.955	1.041	0.900
Male	1.207	0.380	3.839	0.749
Body mass index	1.238	1.057	1.451	0.008
Operative time	1.003	0.991	1.014	0.651
Fluoroscopy time	1.056	1.008	1.106	0.022
Right jugular vein puncture	1.357	0.141	13.036	0.791
Atrial fibrillation	0.898	0.130	6.223	0.913

*CI* confidential interval

## Discussion

### Major findings

This study revealed the following key findings: (1) the radiation dose per procedure to the first operator, measured using real-time personal dosimetry, was generally low during CA; (2) higher patient BMI and longer fluoroscopy time were independent predictors of increased radiation exposure to the operator; and (3) operator radiation exposure was markedly elevated in a case requiring additional interventions to manage complications. These findings highlight the value of real-time personal dosimetry in accurately assessing operator exposure on a per-procedure basis.

### Implications for radiation safety in women and pregnant operators

According to the International Commission on Radiological Protection, the recommended whole-body effective dose limit is 100 mSv over five years and 50 mSv in any single year [11, 12]. For women healthcare workers, the effective dose limit is 5 mSv over three months, and for pregnant workers, the fetal dose limit is 1 mSv for the entire pregnancy [6]. Given these stricter thresholds, especially for women of reproductive age and pregnant operators, real-time radiation monitoring offers a practical advantage by enabling short-term awareness of individual procedural exposure. This real-time awareness may help individual operators—particularly women in their reproductive years—make informed decisions about their participation in specific procedures, contributing to a safer and more inclusive work environment.

In our study, 60% of the first operators were women in their reproductive years, and the median exposure per procedure was 2 µSv, suggesting that routine CA procedures involve relatively low operator exposure. However, a case of cardiac tamponade in this cohort resulted in a 50 µSv exposure to the operator—approximately 25 times the median dose—highlighting the potential for significant radiation exposure in rare but critical situations. To illustrate the unique value of real-time dosimetry, we highlighted a case in which the operator's radiation exposure markedly increased during pericardiocentesis. Such sudden exposure peaks would not have been captured by delayed cumulative dosimetry, underscoring the importance of real-time feedback in managing intra-procedural radiation risks in complex and/or unexpected situations. Unlike conventional dosimeters, which provide delayed cumulative data, real-time dosimeters offer immediate feedback, supporting real-time decision-making and potentially improving safety in such high-stakes scenarios. In addition to these clinical benefits, the device used in our study (Mydose Mini, PDM-117, Hitachi-Aloka) was compact, nonintrusive, and easy to wear under the lead apron. It requires no special setup and has already been implemented in clinical settings as a reliable tool for real-time intra-procedural monitoring [8, 9]. These features make it practical for routine use, especially in high-volume electrophysiology laboratories with diverse operator profiles [3–5]. The proportion of women medical students is steadily increasing, and the number of women pursuing careers in cardiology is expected to grow [4, 5, 13, 14]. Therefore, it is increasingly important to provide accurate knowledge [15] and reliable tools for assessing radiation exposure during interventional cardiovascular procedures. Real-time dosimetry can serve not only as a safety measure but also as a means of reducing uncertainty and anxiety regarding radiation risks. By promoting a clear, evidence-based understanding of actual exposure levels, more women physicians may feel reassured and empowered to confidently engage in interventional cardiology without undue concern.

To further clarify the relative advantages and limitations of each dosimetry method, we summarized the key differences between real-time and conventional dosimeters in Table 4. This comparison may aid institutions in selecting appropriate monitoring strategies tailored to the needs of their operators, particularly those with heightened radiation sensitivity.

**Table 4:** Advantages and limitations of real-time dosimeter and standard dosimeter

	Real-time dosimeter	Standard dosimeter
Measurement timing	Immediate, per procedure	Cumulative, typically monthly
Feedback to operator	Real-time feedback during procedure	Delayed, not available during procedure
Precision per procedure	High (procedure-specific)	Low (data averaged over multiple procedures)
Utility in complications	Detects sudden increases in exposure	Cannot detect timing-specific spikes
Ease of interpretation	Requires real-time monitoring setup	Simple summary report
Radiation awareness support	Enhances operator awareness and behavior	Limited educational or behavioral impact
Suitability for risk assessment	Suitable for individual short-term risk assessment	Suitable for long-term cumulative dose tracking
Cost and availability	Higher cost; not universally available	Widely available and cost-effective
Regulatory compliance	Not yet a standard for regulatory reporting	Required for legal dose reporting

### Patient and procedural factors affecting radiation exposure

Our findings are consistent with previous reports indicating that higher patient BMI [16–18] and prolonged fluoroscopy time [19] are risk factors for increased radiation exposure. However, despite a clear correlation between patient BMI and operator radiation dose, no linear relationship was observed between patient BMI and operator radiation dose. This suggests that factors beyond BMI, such as operator technique, shielding practices, and procedural complexity, may also influence the radiation exposure for operator—further underscoring the utility of real-time monitoring in detecting unexpected increases.

Although AF was the most common indication for CA, and cryoballoon procedures are often associated with higher radiation doses, [20, 21] we found no significant difference in operator exposure between procedures for AF and non-AF, or between cryoballoon and radiofrequency ablation. Notably, the highest operator exposure in our study occurred not during ablation itself, but during fluoroscopy-guided pericardiocentesis for cardiac tamponade. While acute procedural complications occur in approximately 2% of CA cases, with cardiac tamponade accounting for 0.55% [3], such events can lead to substantial increases in radiation exposure, regardless of patient BMI or fluoroscopy time.

Right jugular vein puncture was associated with increased radiation exposure to both operators and patients. Previous studies have shown that this may be due to the operator's closer proximity to the X-ray source and reduced shielding effectiveness [22]. In our procedures, right jugular access was frequently used to place catheters in the coronary sinus. Emerging techniques, including ICE and 3D mapping, may allow for safer navigation of these catheters with reduced fluoroscopy, and should be considered for radiation reduction [6]. Alternatively, the femoral approach for coronary sinus catheter insertion should be considered in future cases from the perspective of radiation safety.

These features make real-time dosimetry particularly useful in high-volume electrophysiology labs, where multiple procedures are performed daily by different operators with varying levels of experience and radiation sensitivity. Real-time monitoring enables immediate recognition of unexpectedly high exposure events, especially during complex or prolonged procedures, and can support timely decisions such as adjusting fluoroscopy technique or rotating operators. Incorporating this approach may contribute to more individualized and adaptive radiation protection strategies in contemporary ablation practice.

### Clinical implications

Although﻿﻿ ﻿﻿the radiation dose per CA procedure was generally low, the potential for abrupt increases due to complications should not be overlooked. Real-time personal dosimetry offers significant advantages over conventional cumulative dosimeters, particularly for radiation-sensitive individuals. As the interventional cardiology field, including catheter ablation, continues to diversify and include more women, practical and individualized radiation protection strategies, including real-time exposure tracking—will be essential. Fostering an environment where operators are well-informed and confident about radiation safety may encourage more women to actively pursue and remain in interventional cardiology without unnecessary hesitation.

### Limitations

This study had several limitations. First, this was a single-center, retrospective study. Second, the lead aprons were different for each individual, and not all operators used the same type or thickness of protective equipment. Third, we accurately measured the operator's radiation exposure using personal real-time dosimetry. However, while real-time monitoring allows for the assessment of exposure per procedure, subsequent changes in exposure trends or long-term monitoring over time remain unclear in this study and warrant further research.

Fourth, although we were able to assess the actual radiation exposure per procedure, we did not evaluate the cumulative dose over time or assess exposure in relation to age-specific thresholds. Therefore, the long-term implications of radiation exposure, particularly in radiation-sensitive operators such as women of reproductive age, could not be addressed. Finally, as our study did not include positional data or operator movement tracking, the potential impact of this factor on operator radiation exposure could not be assessed. 

## Abbreviations

AF: Atrial fibrillation
CA: Catheter ablation

## References

[1] Holmes DR Jr, Alkhouli M (2020) Past, present, and future of interventional cardiology. J Am Coll Cardiol 75:2738–2743 32466890 10.1016/j.jacc.2020.03.066

[2] Wunderle KA, Chung MK, Rayadurgam S, Miller MA, Obuchowski NA, Lindsay BD (2019) Occupational and patient radiation doses in a modern cardiac electrophysiology laboratory. J Interv Card Electrophysiol 56:183–190 30280302 10.1007/s10840-018-0462-8

[3] Kusano K, Inoue K, Kanaoka K, Miyamoto K, Okumura Y, Iwasaki YK, Satomi K, Takatsuki S, Nakamura K, Iwanaga Y, Yamane T, Shimizu W (2024) The Japanese Catheter Ablation Registry (J-AB): annual report in 2022. J Arrhythm 40:1053–1058 39416252 10.1002/joa3.13141 PMC11474523

[4] Tamirisa KP, Alasnag M, Calvert P, Islam S, Bhardwaj A, Pakanati K, Zieroth S, Razminia M, Dalal AS, Mamas M, Russo AM, Kort S (2024) Radiation exposure, training, and safety in cardiology. JACC Adv 3:100863 38939686 10.1016/j.jacadv.2024.100863 PMC11198606

[5] Yong CM, Abnousi F, Rzeszut AK, Douglas PS, Harrington RA, Mehran R, Grines C, Altin SE, Duvernoy CS (2019) Sex differences in the pursuit of interventional cardiology as a subspecialty among cardiovascular fellows-in-training. JACC Cardiovasc Interv 12:219–228 30660463 10.1016/j.jcin.2018.09.036

[6] Fetterly KA, Schueler BA, Mihailovic JM, Fiedler JH, Sturchio GM, Cabalka A, Best PJM, Gulati R, Guerrero M (2024) Radiation exposure and protection for (assumed) pregnant interventional cardiologists and electrophysiologists. J Soc Cardiovasc Angiogr Interv 3:102239 39575215 10.1016/j.jscai.2024.102239 PMC11576368

[7] Durán A, Hian SK, Miller DL, Le Heron J, Padovani R, Vano E (2013) Recommendations for occupational radiation protection in interventional cardiology. Catheter Cardiovasc Interv 82:29–42 23475846 10.1002/ccd.24694

[8] Nikko Radicom Co. L. (2009) Electronic pocket dosimeter. Nikko Radicom Co., Ltd., Tokyo. Available from: https://www.nikko-radicom.com/product/pdf/01h.pdf

[9] Mori H, KK, Ichikawa K (2007) Estimation of personal dose based on the dependent calibration of personal dosimeters in interventional radiology. Jpn J Radiol Technol 63:852–861 10.6009/jjrt.63.852 17917349

[10] Katsurada M, Izumo T, Nagai Y, Chavez C, Kitagawa M, Torii J, Iwase T, Aso T, Tsuchida T, Sasada S (2014) The dose and risk factors for radiation exposure to medical staff during endobronchial ultrasonography with a guide sheath for peripheral pulmonary lesions under X-ray fluoroscopy. Jpn J Clin Oncol 44:257–262 24470585 10.1093/jjco/hyt224

[11] Stewart FA, Akleyev AV, Hauer-Jensen M, Hendry JH, Kleiman NJ, Macvittie TJ, Aleman BM, Edgar AB, Mabuchi K, Muirhead CR, Shore RE, Wallace WH (2012) ICRP publication 118: ICRP statement on tissue reactions and early and late effects of radiation in normal tissues and organs–threshold doses for tissue reactions in a radiation protection context. Ann ICRP 41:1–322 22925378 10.1016/j.icrp.2012.02.001

[12] Kozuma K, Chikamori T, Hashimoto J, Honye J, Ikeda T, Ishiwata S, Kato M, Kondo H, Matsubara K, Matsumoto K, Matsumoto N, Motoyama S, Obunai K, Sakamoto H, Soejima K, Suzuki S, Abe K, Amano H, Hioki H, Iimori T, Kawai H, Kosuge H, Nakama T, Suzuki Y, Takeda K, Ueda A, Yamashita T, Hirao K, Kimura T, Nagai R, Nakamura M, Shimizu W, Tamaki N (2022) JCS 2021 guideline on radiation safety in cardiology. Circ J 86:1148–1203 35466155 10.1253/circj.CJ-21-0379

[13] Walsh MN (2018) Women as leaders in cardiovascular medicine. Clin Cardiol 41:269–273 29485719 10.1002/clc.22920 PMC6489735

[14] Nishizaki F, Shimbo M, Fukue N, Matsumoto C, Noma S, Ohno-Urabe S, Kamiya CA, Kanki S, Ide T, Izawa H, Taniguchi T, Nakayama A, Kobayashi Y (2023) National survey identifying the factors affecting the career development of cardiologists in Japan. Circ J 87:1219–1228 37380440 10.1253/circj.CJ-23-0063

[15] Capranzano P, Kunadian V, Mauri J, Petronio AS, Salvatella N, Appelman Y, Gilard M, Mikhail GW, Schüpke S, Radu MD, Vaquerizo B, Presbitero P, Morice MC, Mehilli J (2016) Motivations for and barriers to choosing an interventional cardiology career path: results from the EAPCI Women Committee worldwide survey. EuroIntervention 12:53–59 26151955 10.4244/EIJY15M07_03

[16] Madder RD, VanOosterhout S, Mulder A, Ten Brock T, Clarey AT, Parker JL, Jacoby ME (2019) Patient body mass index and physician radiation dose during coronary angiography. Circ Cardiovasc Interv 12:e006823 30599769 10.1161/CIRCINTERVENTIONS.118.006823

[17] Koh Y, Vogrin S, Noaman S, Lam S, Pham R, Clark A, Biffin L, Hanson LB, Bloom JE, Stub D, Brennan AL, Reid C, Dinh DT, Lefkovits J, Cox N, Chan W (2022) Effect of different anthropometric body indexes on radiation exposure in patients undergoing cardiac catheterisation and percutaneous coronary intervention. Tomography 8:2256–2267 36136885 10.3390/tomography8050189 PMC9498890

[18] Crowhurst J, Savage M, Hay K, Murdoch D, Aroney N, Dautov R, Walters DL, Raffel OC (2022) Impact of patient BMI on patient and operator radiation dose during percutaneous coronary intervention. Heart Lung Circ 31:372–382 34654649 10.1016/j.hlc.2021.08.019

[19] Rosenthal LS, Mahesh M, Beck TJ, Saul JP, Miller JM, Kay N, Klein LS, Huang S, Gillette P, Prystowsky E, Carlson M, Berger RD, Lawrence JH, Yong P, Calkins H (1998) Predictors of fluoroscopy time and estimated radiation exposure during radiofrequency catheter ablation procedures. Am J Cardiol 82:451–458 9723632 10.1016/s0002-9149(98)00356-7

[20] Straube F, Dorwarth U, Ammar-Busch S, Peter T, Noelker G, Massa T, Kuniss M, Ewertsen NC, Chun KR, Tebbenjohanns J, Tilz R, Kuck KH, Ouarrak T, Senges J, Hoffmann E (2016) First-line catheter ablation of paroxysmal atrial fibrillation: outcome of radiofrequency vs. cryoballoon pulmonary vein isolation. Europace 18:368–375 26504108 10.1093/europace/euv271

[21] Hoffmann E, Straube F, Wegscheider K, Kuniss M, Andresen D, Wu LQ, Tebbenjohanns J, Noelker G, Tilz RR, Chun JKR, Franke A, Stellbrink C, Garcia-Alberola A, Dorwarth U, Metzner A, Ouarrak T, Brachmann J, Kuck KH, Senges J (2019) Outcomes of cryoballoon or radiofrequency ablation in symptomatic paroxysmal or persistent atrial fibrillation. Europace 21:1313–1324 31199860 10.1093/europace/euz155 PMC6735953

[22] Chen W, Yao Y, Zhang S, He DS (2011) Comparison of operator radiation exposure during coronary sinus catheter placement via the femoral or jugular vein approach. Europace 13:539–542 21252193 10.1093/europace/euq515

